# Clinical Application of CHA_2_DS_2_-VASc versus GRACE Scores for Assessing the Risk of Long-term Ischemic Events in Atrial Fibrillation and Acute Coronary Syndrome or PCI

**DOI:** 10.31083/j.rcm2305168

**Published:** 2022-05-11

**Authors:** Ran Mo, Yan-min Yang, Han Zhang, Ni Suo, Jing-yang Wang, Si-qi Lyu

**Affiliations:** ^1^Emergency Center, Fuwai Hospital, National Center for Cardiovascular Diseases, Chinese Academy of Medical Sciences and Peking Union Medical College, 100037 Beijing, China; ^2^National Clinical Research Center of Cardiovascular Diseases, Fuwai Hospital, National Center for Cardiovascular Diseases, Chinese Academy of Medical Sciences and Peking Union Medical College, 100037 Beijing, China

**Keywords:** atrial fibrillation, acute coronary syndrome, percutaneous coronary intervention, GRACE, CHA_2_DS_2_-VASc score

## Abstract

**Background:**

Early risk stratification of patients with atrial fibrillation (AF) and acute coronary syndrome (ACS) or undergoing percutaneous coronary intervention (PCI) has relevant implication for individualized management strategies. The ${\mathrm{CHA}}_{2}$${\mathrm{DS}}_{2}$-VASc and GRACE ACS risk model are well-established risk stratification systems. We aimed to assess their prognostic performance in AF patients with ACS or PCI.

**Methods:**

Consecutive patients with AF and ACS or referred for PCI were prospectively recruited and followed up for 3 years. The primary endpoint was major adverse cardiovascular and cerebrovascular events (MACCEs), including cardiovascular mortality, myocardial infarction, ischemic stroke, systemic embolism and ischemia-driven revascularization.

**Results:**

Higher ${\mathrm{CHA}}_{2}$${\mathrm{DS}}_{2}$-VASc (HR [hazard ratio] 1.184, 95% CI 1.091–1.284) and GRACE at discharge score (HR 1.009, 95% CI 1.004–1.014) were independently associated with increased risk of MACCEs. The ${\mathrm{CHA}}_{2}$${\mathrm{DS}}_{2}$-VASc (c-statistics: 0.677) and GRACE at discharge (c-statistics: 0.699) demonstrated comparable discriminative capacity for MACCEs (*p* = 0.281) while GRACE at admission provided relatively lower discrimination (c-statistics: 0.629, *p* vs. ${\mathrm{CHA}}_{2}$${\mathrm{DS}}_{2}$-VASc = 0.041). For predicting all-cause mortality, three models displayed good discriminative capacity (c-statistics: 0.750 for ${\mathrm{CHA}}_{2}$${\mathrm{DS}}_{2}$-VASc, 0.775 for GRACE at admission, 0.846 for GRACE at discharge). A significant discrimination improvement of GRACE at discharge compared to ${\mathrm{CHA}}_{2}$${\mathrm{DS}}_{2}$-VASc was detected (NRI = 45.13%).

**Conclusions:**

In the setting of coexistence of AF and ACS or PCI, ${\mathrm{CHA}}_{2}$${\mathrm{DS}}_{2}$-VASc and GRACE at discharge score were independently associated with an increased risk of MACCEs. The GRACE at discharge performed better in predicting all-cause mortality.

## 1. Introduction 

Atrial fibrillation (AF) is the most common sustained cardiac arrhythmia, occurring in 2%–4% of the general population with increasing prevalence among the elderly [1]. It is predicted that 10%–15% AF patients will require percutaneous coronary intervention (PCI) for coronary artery disease (CAD) during their life, while patients with AF and acute coronary syndrome (ACS) will be more likely to experience adverse outcomes than ACS patients without AF [2, 3]. Concomitant risks of subsequent ischemic events, in-stent thrombosis and treatment-related bleeding need to be carefully considered before initiating appropriate antithrombotic therapy.

Several risk clinical scores have been proven to enhance the assessment of thrombo-embolic risk in AF. ${\mathrm{CHA}}_{2}$${\mathrm{DS}}_{2}$-VASc score [congestive heart failure (CHF), hypertension, age ≥75 years, diabetes mellitus, stroke, vascular disease, age 65–74 years, sex category (female)] is a recognized tool to stratify stroke risk and recommended by guidelines [4, 5]. Several studies have showed that a higher ${\mathrm{CHA}}_{2}$${\mathrm{DS}}_{2}$-VASc score was independently associated with a poor outcome in CAD patients with sinus rhythm [6, 7, 8].

Early risk stratification is also important for ACS patients to help clinicians to determine prognosis and therefore guide management strategies. A number of prognostic models have been developed including Global Registry of Acute Coronary Events (GRACE) [9], thrombolysis in myocardial infarction (TIMI) [10], and platelet glycoprotein IIb/IIIa in unstable angina: receptor suppression using Integrilin therapy (PRUSUIT) [11]. Among these, the GRACE risk score has been externally validated and proved to display the best discriminative performance [12]. The GRACE score at admission [age, systolic blood pressure (SBP), heart rate, serum creatinine, cardiac arrest at admission, elevated cardiac biomarkers, ST-segment deviation and Killip class at presentation] is established and widely accepted for assessing death or myocardial infarction from admission to six months after discharge [13]. Meanwhile, another prediction model, the GRACE score at discharge [age, history of CHF, history of myocardial infarction (MI), heart rate, SBP, ST-segment depression, serum creatinine, elevated cardiac enzymes and no in-hospital PCI] also derived by the implemented GRACE registry, has been established a robust tool for predicting 6-month post-discharge mortality in patients with ACS [14].

For patients with AF and ACS, it still remains unclear whether ${\mathrm{CHA}}_{2}$${\mathrm{DS}}_{2}$-VASc or GRACE score may be useful to assess the risk of major adverse cardiovascular and cerebrovascular events (MACCEs) and thus guide the anticoagulant regimens. Therefore, in the present study we aim to compare the prognostic values of ${\mathrm{CHA}}_{2}$${\mathrm{DS}}_{2}$-VASc, GRACE score at admission and GRACE score at discharge in predicting long-term MACCEs in patients with AF and ACS or undergoing PCI.

## 2. Materials and Methods

### 2.1 Study Population and Data Collection

This is an observational, prospective, single-center registry. From January 2017 to December 2018, a total of 1408 patients with AF (new-onset, paroxysmal, persistent, long-standing or permanent) who were diagnosed with ACS or referred for PCI were consecutively enrolled in the present study. All participants aged <18 years and patients unable/unwilling to finish the follow-up were excluded from the analysis. Demographic characteristics, medical history, clinical exams, laboratory tests and discharge medications were collected from the medical records. The classification of AF was in accordance with 2020 ESC guideline [5]. Hypertension was defined by self-reported and diabetes mellitus (DM) was defined by either self-reported or hemoglobin A1c ≥6.5%. Information of heart failure status was obtained by viewing medical records retrospectively. The laboratory results at admission included hemoglobin (Hb), serum potassium, creatinine, estimated glomerular filtration rate (eGFR), increase in cardiac troponin I (cTNI), N-terminal pro-B-type natriuretic peptide (NT-proBNP), low-density lipoprotein cholesterol (LDL-C), international normalized ratio (INR) and HbA1c. Left ventricular ejection fraction (LVEF) was measured by experienced physicians using echo-cardiography.

Patients were categorized into three risk groups according to the GRACE score at admission (low: ≤108, intermediate: 109–140, high: >140), the GRACE score at discharge (low: ≤88, intermediate: 89–118, high: >118) and ${\mathrm{CHA}}_{2}$${\mathrm{DS}}_{2}$-VASc score (low: 1–2, intermediate: 3–4, high: >4) respectively.

### 2.2 Endpoints

Cardiovascular (CV) death was adjudicated as any death with a demonstrable cardiovascular cause or any death that was not clearly attributable to a noncardiovascular cause. MI (myocardial infarction)was defined according to the third universal definition of MI [15]. Ischemic stroke was adjudicated as an episode of neurological dysfunction caused by focal cerebral, spinal, or retinal infraction and transient ischemic attack (TIA) was defined as focal cerebral ischemic event with symptoms lasting <24 hours. Ischemia-driven coronary revascularization included all coronary revascularization during follow-up that were performed in the context of MI and those for worsening symptoms in combination with evidence of myocardial ischemia. Systemic embolism was defined as new acute limb ischemia or objective evidence of sudden loss of perfusion of a limb or an organ. Every adverse event was carefully reviewed by an independent clinical event adjudication committee.

The primary outcome of interest was MACCEs defined as cardiovascular (CV) mortality, myocardial infarction (MI), ischemic stroke or TIA, systemic embolism and ischemia-driven revascularization in follow-up and these events were analyzed individually. All-cause mortality was analyzed as a secondary end-point. The primary safety objective was a composite of major bleeding according to the Thrombolysis in Myocardial Infarction (TIMI) criteria [16] or bleeding in need of medical attention.

Follow-up by telephone interviews or clinic visits were scheduled every 6 months lasting for 36 months. Every adverse event or bleeding was carefully reviewed by an independent clinical event adjudication committee. The study was approved by the ethics committee of Fuwai Hospital and was conducted in accordance with the Declaration of Helsinki. All subjects provided written consent form before participation.

### 2.3 Statistical Analysis

For baseline characteristics, categorical variables were displayed as frequencies (percentages), and continuous variables were expressed as means ± standard deviations (SD) or medians with interquartile range (IQR) if they were not normally distributed. Normality was evaluated using the Shapiro-Wilk W-test. Continuous variables were compared using independent Student’s *t*-test or Mann-Whitney test as appropriate while categorical variables using Pearson’s chi-squared test or Fisher’s exact test. Risk predictive models were analyzed both as a continuous and class variable. In order to adjust the effects by potential confounding risk factors, Cox proportional hazards models were used to perform multivariable analysis. Models included GRACE, ${\mathrm{CHA}}_{2}$${\mathrm{DS}}_{2}$-VASc score and were adjusted for all the variables not included in the three risk models which showed an independent association with a *p* value < 0.10 in the univariable analysis. A backward stepwise selection algorithm was used. The results were displayed as hazards ratios (HRs) and their 95% confidence intervals (95% CI). Kaplan-Meier curves were generated and statistical differences were assessed by log-rank test (after Bonferroni correction α = 0.0167) in clinical endpoints between subgroups.

Receiver-operating curves (ROC) and c-statistics were constructed for MACCE and all-cause mortality to compare the discrimination performance of the three models. The statistical difference of c-statistics was evaluated through the Delong method and the net classification improvement (NRI) was further calculated.

The software package SPSS version 25.0 (IBM Corporation, New York, NY, USA) and R version 4.1.2 (R Core Team, Vienna, Austria) were utilized for statistical analysis. All statistical tests were 2-tailed, with a *p* value < 0.05 considered statistically significant.

## 3. Results

### 3.1 Baseline Characteristics

A total of 1408 patients were included and their baseline characteristics categorized by outcomes were displayed in Table 1. The mean age was 67.3 ± 9.4 years. Nearly three quarters of the patients were male (72.9%) and 471 (33.5%) were admitted through emergency department. Previously to the inclusion of the study, 419 (29.8%) had received PCI therapy. By the time of recruitment, 302 (21.4%) patients were complicated with congestive heart failure, and the proportions for hypertension, DM and previous stroke/TIA were 77.3%, 42.6% and 25.2% respectively. At the time of inclusion, more than half of participants (56.5%) suffered paroxysmal AF and 975 (69.3%) suffered ACS. The median value of NT-proBNP was 766.95 pg/mL (IQR 234.78–2122.95) and the average LVEF was 56.3 ± 10.0%. At the time of discharge, 1147 (81.5%) patients were prescribed aspirin while 628 (48.4%) patients received anticoagulant therapy including warfarin, Rivaroxaban or Dabigatran.

**Table 1.** **Baseline characteristics and medical therapies of the study population according with primary and secondary outcome**.

Variable	Total (n = 1408)	MACCE		*p* value	All-cause death		*p* value
		No (n = 1188)	Yes (n = 220)		No (n = 1267)	Yes (n = 141)	
Age (years)	67.3 ± 9.4	67.0 ± 9.3	69.3 ± 9.8	0.001	66.7 ± 9.2	73.1 ± 9.6	<0.001
Male , n (%)	1027 (72.9)	890 (74.9)	137 (62.3)	<0.001	935 (73.8)	92 (62.5)	0.036
Emergency presentation, n (%)	471 (33.5)	370 (31.1)	101 (45.9)	<0.001	381 (30.1)	90 (63.8)	<0.001
Vital signs							
BMI (kg/$m^{2}$)	25.17 ± 5.08	25.26 ± 4.96	24.70 ± 5.70	0.131	25.39 ± 3.76	23.20 ± 7.10	<0.001
SBP (mmHg)	130.4 ± 19.5	130.7 ± 19.2	128.8 ± 21.5	0.209	130.9 ± 19.0	126.2 ± 23.6	0.025
DBP (mmHg)	77.1 ± 11.4	77.1 ± 11.2	76.8 ± 12.3	0.738	77.2 ± 11.2	75.7 ± 12.7	0.178
Resting heart rate (bpm)	78 (64–82)	70 (64–80)	75 (66–91)	<0.001	70 (64–80)	78 (67–95)	<0.001
Medical history, n (%)							
Myocardial infarction	397 (28.2)	310 (26.1)	87 (39.5)	<0.001	324 (25.6)	73 (51.8)	<0.001
PCI	419 (29.8)	351 (29.5)	68 (30.9)	0.689	370 (29.2)	49 (34.8)	0.175
Heart failure	302 (21.4)	209 (17.6)	93 (42.3)	<0.001	230 (18.2)	72 (51.1)	<0.001
Hypertension	1088 (77.3)	909 (76.5)	179 (81.4)	0.136	977 (77.1)	111(78.7)	0.751
Hyperlipidemia	1037 (73.7)	872 (73.4)	165 (75.0)	0.677	930 (73.4)	107 (75.9)	0.614
Diabetes	600 (42.6)	490 (41.2)	110 (50.0)	0.018	527 (41.6)	73 (51.8)	0.025
Stroke/TIA	355 (25.2)	278 (23.4)	77 (35.0)	<0.001	305 (24.1)	50 (35.5)	0.004
Chronic kidney disease	187 (13.3)	133 (11.2)	54 (24.5)	<0.001	131 (10.3)	56 (39.7)	<0.001
AF pattern, n (%)				<0.001			0.330
First diagnosed	106 (7.5)	87 (7.3)	19 (8.6)		96 (7.6)	10 (7.1)	
Paroxysmal	795 (56.5)	701 (59.0)	94 (42.7)		725 (57.2)	70 (49.6)	
Persistent	462 (32.8)	364 (30.6)	98 (44.5)		406 (32.0)	56 (39.7)	
Long-standing persistent	41 (2.9)	33 (2.8)	8 (3.6)		37 (2.9)	4 (2.8)	
Permanent	4 (0.3)	3 (0.3)	1 (0.5)		3 (0.2)	1 (0.7)	
Diagnosis for CAD, n (%)				0.001			<0.001
SCAD	433 (30.8)	379 (31.9)	54 (24.5)		414 (32.7)	18 (13.5)	
Unstable angina	471 (33.5)	402 (33.8)	69 (31.4)		436 (34.4)	35 (24.8)	
STEMI	204 (14.5)	176 (14.8)	28 (12.7)		171 (13.5)	33 (23.4)	
NSTEMI	300 (21.3)	231 (19.4)	69 (31.4)		246 (19.4)	54 (38.3)	
Laboratory test							
Hemoglobin (g/dL)	14.25 ± 1.92	14.33±1.83	13.82±2.27	<0.001	14.38 ± 1.83	13.14 ± 2.32	<0.001
Serum potassium (mmol/L)	4.18 ± 0.45	4.17 ± 0.45	4.23 ± 0.49	0.096	4.18 ± 0.45	4.21 ± 0.51	0.363
Creatinine (mg/dL)	1.07 ± 0.31	1.05 ± 0.29	1.16 ± 0.40	<0.001	1.04 ± 0.29	1.30 ± 0.44	<0.001
eGFR (mL/min/1.73 $m^{2}$)	77.96 ± 22.74	79.38 ± 22.47	70.31 ± 22.69	<0.001	79.74 ± 22.10	61.93 ± 22.13	<0.001
cTNI elevation	0.3 (0.0–3.6)	0.3 (0.0–3.2)	1.1 (0.0–7.4)	0.052	0.3 (0.0–3.0)	2.0 (0.3–23.3)	<0.001
NT-proBNP (pg/mL)	766.95 (234.78–2122.95)	619.05 (205.03–1691.88)	1920.5 (704.28–5004.38)	<0.001	630.40 (210.90–1692.0)	3334.4 (1550.20–9368.80)	<0.001
LDL-C (mg/dL)	89.71 ± 33.64	89.71 ± 34.03	90.87 ± 32.48	0.620	89.71 ± 33.64	88.94 ± 34.03	0.766
INR	1.15 ± 0.47	1.12 ± 0.35	1.28 ± 0.84	0.008	1.12 ± 0.36	1.34 ± 0.99	0.011
HbA1c (%)	6.63 ± 1.20	6.59 ± 1.18	6.86 ± 1.24	0.005	6.62 ± 1.19	6.78 ± 1.22	0.159
LVEF (%)	56.3 ± 10.0	57.2 ± 9.3	51.5 ± 12.4	<0.001	57.4 ± 9.2	46.6 ± 12.1	<0.001
Medications, n (%)							
Aspirin	1147 (81.5)	996 (83.8)	151 (68.6)	<0.001	1053 (83.1)	94 (66.7)	<0.001
Clopidogrel	1257 (89.3)	1054 (88.7)	203 (92.3)	0.124	1132 (89.3)	125 (88.7)	0.801
Ticagrelor	118 (8.4)	111 (9.3)	7 (3.2)	0.001	114 (9.0)	4 (2.8)	0.010
Anticoagulant therapy	682 (48.4)	528 (44.4)	154 (70.0)	<0.001	612 (48.3)	70 (49.6)	0.790
Statin	1375 (97.7)	167 (98.2)	208 (94.5)	0.003	1247 (98.4)	128 (90.8)	<0.001
ACEi or ARB	898 (63.8)	770 (64.8)	128 (58.2)	0.067	828 (65.4)	70 (49.6)	<0.001
Diuretics	550 (39.1)	425 (35.8)	125 (56.8)	<0.001	447 (35.3)	103 (73.0)	<0.001
β-blocker	1210 (85.9)	1017 (85.6)	193 (87.7)	0.460	1090 (86.0)	120 (85.1)	0.798
${\mathrm{CHA}}_{2}$${\mathrm{DS}}_{2}$-VASc	3.7 ± 1.8	3.6 ± 1.7	4.6 ± 1.8	<0.001	3.6 ± 1.7	5.1 ± 1.8	<0.001
GRACE at admission	126 ± 36	123 ± 33	142 ± 43	<0.001	122 ± 33	161 ± 42	<0.001
GRACE at discharge	108 ± 33	105 ± 31	126 ± 36	<0.001	104 ± 30	144 ± 32	<0.001

MACCE, major adverse cardiovascular and cerebrovascular events; BMI, body mass index; SBP, systolic blood pressure; DBP, diastolic blood pressure; PCI, percutaneous coronary intervention; TIA, transient ischemic attack; AF, atrial fibrillation; CAD, coronary artery disease; SCAD, stable coronary artery diseases; eGFR, estimated glomerular filtration fraction; NT-proBNP, N-terminal pro-B-type natriuretic peptide; LDL-C, low-density lipoprotein cholesterol; INR, international normalized ratio; LVEF, left ventricular ejection fraction.

The mean value of ${\mathrm{CHA}}_{2}$${\mathrm{DS}}_{2}$-VASc score was 3.7 ± 1.8 and the distribution of ${\mathrm{CHA}}_{2}$${\mathrm{DS}}_{2}$-VASc was displayed in **Supplementary Table 1**. The average scores of GRACE score at admission and GRACE score at discharge were 126 ± 36 and 108 ± 33 respectively.

### 3.2 Clinical Outcomes and Multivariable Analysis

The clinical outcomes classified by GRACE and ${\mathrm{CHA}}_{2}$${\mathrm{DS}}_{2}$-VASc subgroups were shown in Table 2. During follow-up, 220 (15.6%) primary outcomes occurred, of which 99 (7.0%) patients suffered CV mortality, 39 (2.8%) suffered MI, 57 (4.0%) experienced stroke or TIA, 56 (4.0%) received coronary revascularization driven by ischemic symptoms and only 6 (0.4%) suffered systemic embolism. 136 (9.7%) all-cause death occurred and 24 (1.7%) patients experienced major bleeding. Based on ${\mathrm{CHA}}_{2}$${\mathrm{DS}}_{2}$-VASc score, 380 (27.0%) patients were at low risk, 584 (41.5%) at intermediate risk and 444 (31.5%) at high risk. Regarding the GRACE score at admission, 515 (36.6%) patients were categorized as low risk, 517 (36.7%) at intermediate risk and 376 (26.7%) at high risk. The rates for GRACE score at discharge were 29.0%, 38.0% and 33.0%.

**Table 2.** **Adverse events at 36 months follow-up according to ${\mathrm{CHA}}_{2}$${\mathrm{DS}}_{2}$-VASc and GRACE score**.

Endpoints		${\mathrm{CHA}}_{2}$${\mathrm{DS}}_{2}$-VASc			GRACE score at admission			GRACE score at discharge		
	Total (n = 1408)	1, 2 (n = 380)	3, 4 (n = 584)	>4 (n = 444)	≤108 (n = 515)	109–140 (n = 517)	>140 (n = 376)	≤88 (n = 408)	89–118 (n = 536)	>118 (n = 464)
MACCE	220 (15.6)	29 (7.6)	79 (13.5)	112 (25.2)	57 (11.1)	69 (13.3)	94 (25.0)	36 (8.8)	69 (12.9)	115 (24.8)
Cardiovascular mortality	99 (7.0)	7 (1.8)	29 (5.0)	63 (14.2)	9 (1.7)	26 (5.0)	64 (17.0)	3 (0.7)	18 (3.4)	78 (16.8)
Myocardial infarction	39 (2.8)	2 (0.5)	11 (1.9)	26 (5.9)	7 (1.4)	11 (2.1)	21 (5.7)	2 (0.5)	14 (2.6)	23 (5.0)
Stroke/TIA	57 (4.0)	6 (1.6)	25 (4.3)	26 (5.9)	20 (3.9)	21 (4.1)	16 (4.3)	16 (3.9)	19 (3.5)	22 (4.7)
Ischemia-driven revascularization	56 (4.0)	14 (3.7)	23 (4.0)	19 (4.3)	27 (5.2)	21 (4.1)	8 (2.2)	17 (4.2)	29 (5.4)	10 (2.2)
Systemic embolism	6 (0.4)	0 (0.0)	5 (0.9)	1 (0.2)	2 (0.4)	3 (0.6)	1 (0.3)	2 (0.5)	1 (0.2)	3 (0.7)
All-cause mortality	136 (9.7)	8 (2.1)	43 (7.4)	85 (19.1)	11 (2.1)	38 (7.4)	89 (23.7)	4 (1.0)	27 (5.0)	105 (22.6)
Major bleeding	24 (1.7)	5 (1.3)	10 (1.7)	9 (2.1)	6 (1.2)	13 (2.5)	5 (1.4)	5 (1.2)	13 (2.4)	6 (1.3)
Minor bleeding	24 (1.7)	2 (0.5)	10 (1.7)	12 (2.7)	3 (0.6)	9 (1.7)	12 (3.2)	1 (0.2)	9 (1.7)	14 (3.1)

Data presented as number of events and 36-month Kaplan-Meier estimates: n (%). Abbreviations: MACCE, major adverse cardiovascular and cerebrovascular events; TIA, transient ischemic attack.

**Fig. 1.**
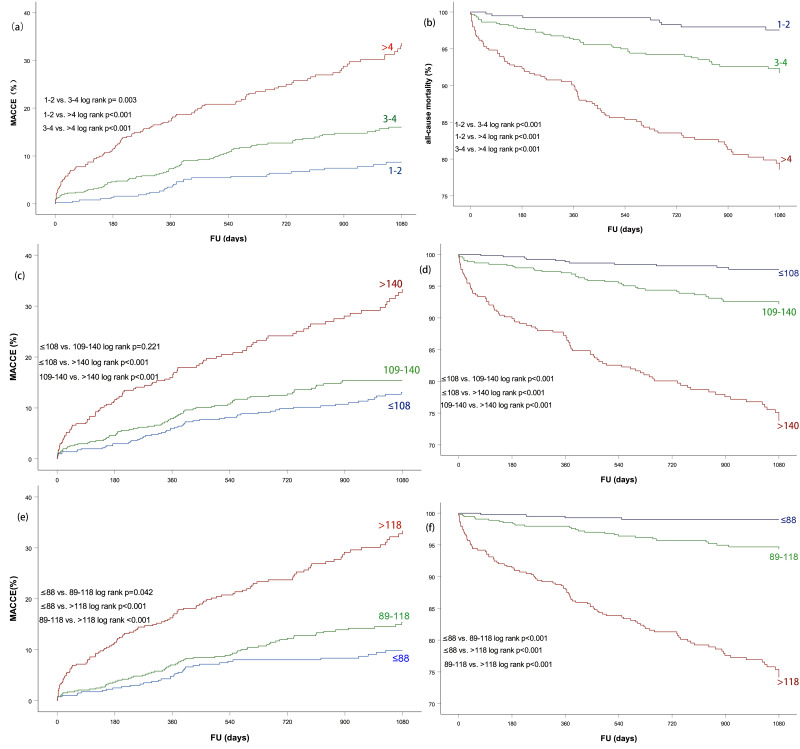
**Kaplan-Meier curves for 36-month adverse events in patients at low, intermediate and high risk based on three risk scores**. (a) ${\mathrm{CHA}}_{2}$${\mathrm{DS}}_{2}$-VASc for MACCEs. (c) GRACE score at admission for MACCEs. (e) GRACE score at discharge for MACCEs. Event free survival from all-cause mortality based on (b) ${\mathrm{CHA}}_{2}$${\mathrm{DS}}_{2}$-VASc. (d) GRACE at admission and (f) GRACE at discharge.

Kaplan-Meier curves for MACCE and all-cause mortality in patients at low, intermediate and high risk based on different scores were plotted in Fig. 1. Higher ${\mathrm{CHA}}_{2}$${\mathrm{DS}}_{2}$-VASc was obviously associated with increased risk of MACCEs and all-cause mortality during follow-up. However, patients presented with GRACE score at admission ≤108 compared to those with GRACE at admission between 109 and 140 gained similar risk of primary outcome (log rank* p *= 0.221). The GRACE at discharge score was also unable to significantly stratify low and intermediate risk in MACCEs (*p* = 0.042 > 0.0167 after Bonferroni correction α). After adjusting potential cofounders including emergency presentation, AF patterns, subtypes of CAD, baseline hemoglobin, INR, HbA1c, NT-proBNP, LVEF, use of aspirin, Ticagrelor and use of coagulant therapy, ${\mathrm{CHA}}_{2}$${\mathrm{DS}}_{2}$-VASc (HR 1.184, 95% CI 1.091–1.284, *p *< 0.001) and GRACE score at discharge (HR 1.009, 95% CI 1.004–1.014, *p *< 0.001) were independent predictors for subsequent MACCEs as continuous variables. Nevertheless, when treated as categorical variables, both ${\mathrm{CHA}}_{2}$${\mathrm{DS}}_{2}$-VASc (low vs. intermediate: HR 1.449, 95% CI 0.935–2.244,* p *= 0.097; low vs. high: HR 2.226, 95% CI 1.436–3.453, *p *< 0.001) and GRACE at discharge (low vs. intermediate: HR 1.268, 95% CI 0.839–1.917, *p* = 0.260; low vs. high: HR 1.631, 95% CI 1.066–2.498, *p* = 0.024) only retained the ability to identify high-risk subgroups. The multivariable analysis revealed that GRACE score at admission was not a predictor for MACCEs. The ${\mathrm{CHA}}_{2}$${\mathrm{DS}}_{2}$-VASc (continuous: HR 1.348, 95% CI 1.216–1.494, *p *< 0.001), GRACE at admission (continuous: HR 1.013, 95% CI 1.008–1.018, *p* = 0.002) and GRACE at discharge (continuous: HR 1.026, 95% CI 1.020–1.032, *p *< 0.001) were strong and independent predictors for all-cause mortality no matter they were treated as continuous or categorical variables. Regarding the safety endpoints, none of the three risk models provided sufficient predictive ability. The detailed relations between three models and outcomes were shown in Table 3.

**Table 3.** **Multivariable analysis of the ${\mathrm{CHA}}_{2}$${\mathrm{DS}}_{2}$-VASc and GRACE scores for the outcomes of MACCE, all-cause mortality and major bleeding**.

		MACCE		All-cause mortality		Major bleeding	
		HR (95% CI)	*p*	HR (95% CI)	*p*	HR (95% CI)	*p*
${\mathrm{CHA}}_{2}$${\mathrm{DS}}_{2}$-VASc (continuous)		1.184 (1.091–1.284)	<0.001	1.348 (1.216–1.494)	<0.001	0.995 (0.770–1.286)	0.972
	1–2	Reference		Reference		Reference	
	3–4	1.449 (0.935–2.244)	0.097	2.878 (1.285–6.446)	0.010	0.933 (0.304–2.860)	0.903
	>4	2.226 (1.436–3.453)	<0.001	5.457 (2.469–12.061)	<0.001	1.082 (0.335–3.497)	0.895
GRACE at admission (continuous)		1.004 (1.000–1.008)	0.061	1.013 (1.008–1.018)	<0.001	0.992 (0.978–1.006)	0.275
	≤108	Reference		Reference		Reference	
	109–140	1.089 (0.755–1.571)	0.648	2.863 (1.450–5.653)	0.002	3.148 (1.000–9.518)	0.050
	>140	1.32 (0.786–2.229)	0.292	5.309 (2.727–10.336)	<0.001	0.966 (0.207–4.510)	0.966
GRACE at discharge (continuous)		1.009 (1.004–1.014)	<0.001	1.026 (1.020–1.032)	<0.001	0.992 (0.977–1.007)	0.300
	≤88	Reference		Reference		Reference	
	89–118	1.268 (0.839–1.917)	0.260	4.219 (1.471–12.101)	0.007	1.573 (0.550–4.498)	0.399
	>118	1.631 (1.066–2.498)	0.024	11.666 (4.177–32.585)	<0.001	0.638 (0.171–2.383)	0.504

Adjusted for emergency presentation, atrial fibrillation patterns, subtypes of coronary artery disease, hemoglobin, NT-proBNP at admission, LVEF, INR, anticoagulant therapy, use of aspirin, use of ticagrelor.

### 3.3 Comparison of Risk Stratification Models

**Fig. 2.**
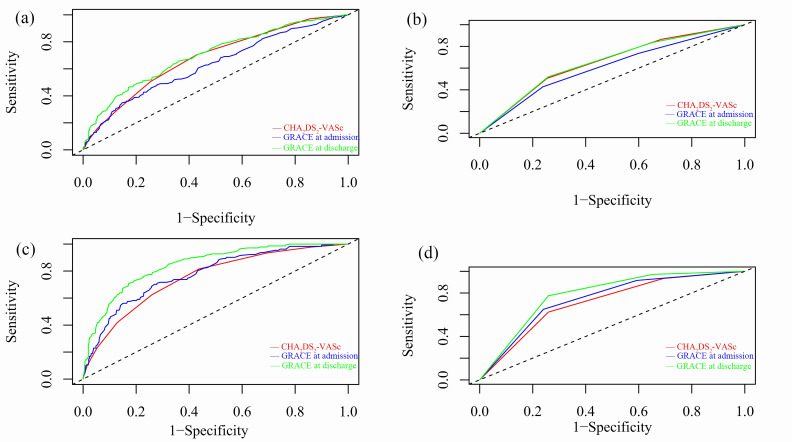
**ROC curves for predicting MACCEs or all-cause mortality during follow-up**. Three scores were treated (a) as continuous variables for MACCEs. (b) as categorical variables for MACCEs. (c) as continuous variables for all-cause mortality. (d) as categorical variables for all-cause mortality.

ROC curves of ${\mathrm{CHA}}_{2}$${\mathrm{DS}}_{2}$-VASc and GRACE scores for predicting MACCEs or all-cause mortality were shown in Fig. 2. The c-statistics and NRI analyses were shown in **Supplementary Table 2**. As continuous variables, the c-statistics for primary outcomes were 0.677 for ${\mathrm{CHA}}_{2}$${\mathrm{DS}}_{2}$-VASc (95% CI 0.647–0.717), 0.629 for GRACE at admission (95% CI 0.585–0.673) and 0.699 for GRACE score at discharge (95% CI 0.659–0.740). The GRACE at discharge and ${\mathrm{CHA}}_{2}$${\mathrm{DS}}_{2}$-VASc scores had comparable prognostic value for MACCEs (*p* = 0.281) but the GRACE score at admission had worse discrimination accuracy compared to ${\mathrm{CHA}}_{2}$${\mathrm{DS}}_{2}$-VASc score (*p* = 0.041, NRI: –13.21%, 95% CI –21.60% to –7.38%). As for all-cause mortality, the ${\mathrm{CHA}}_{2}$${\mathrm{DS}}_{2}$-VASc score (c-statistics: 0.750, 95% CI 0.705–0.794) proved to have similar risk stratification as GRACE score at admission (c-statistics: 0.775, 95% CI 0.732–0.818). Nevertheless, the GRACE at discharge achieved statistically stronger discrimination ability (c-statistics: 0.846, 95% CI 0.813–0.880) compared to ${\mathrm{CHA}}_{2}$${\mathrm{DS}}_{2}$-VASc (NRI: 45.13%, *p *< 0.001).

### 3.4 Subgroup Analyses

**Fig. 3.**
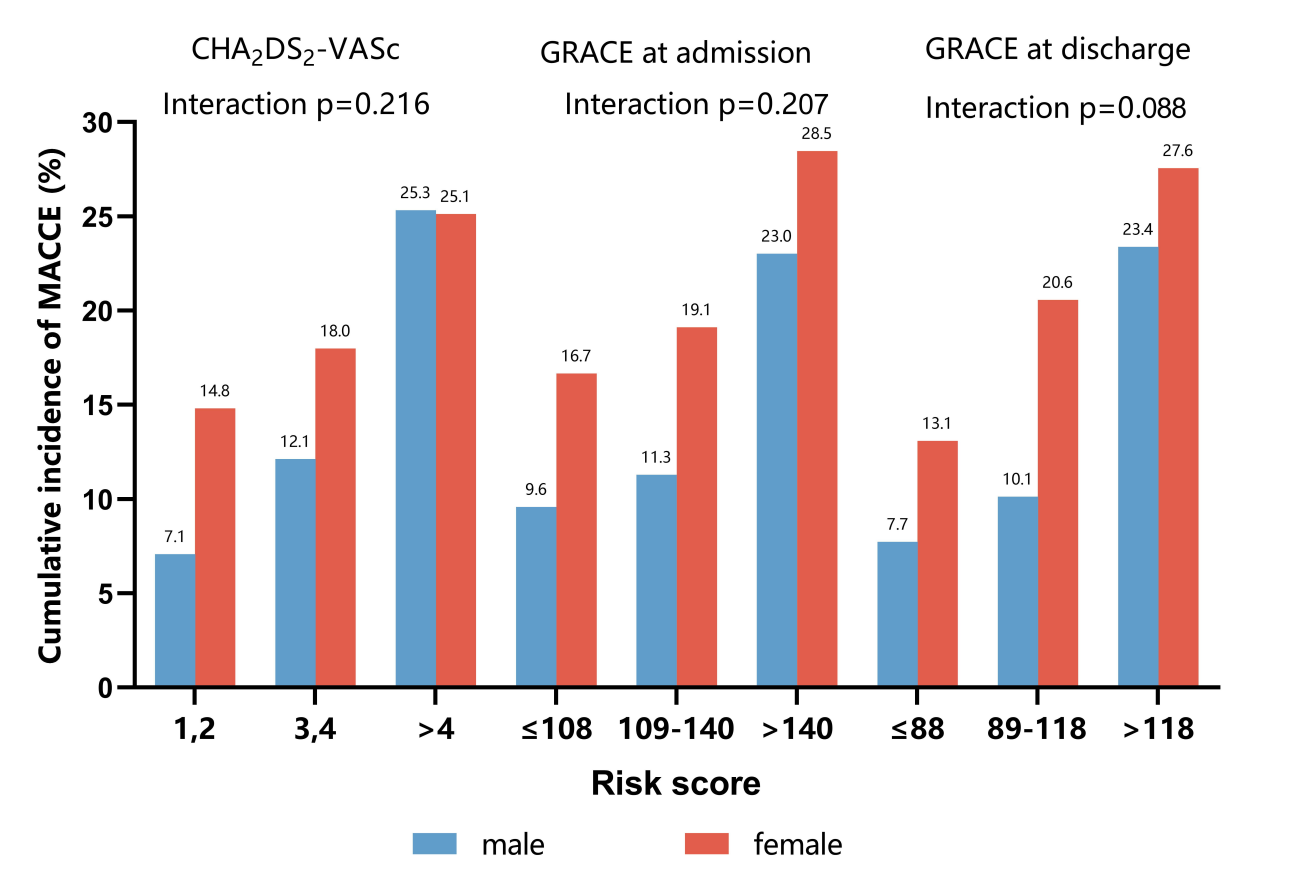
**Cumulative incidence of MACCEs according to sex during 36 months follow-up**. The predictive performance of ${\mathrm{CHA}}_{2}$${\mathrm{DS}}_{2}$-VASc, GRACE at admission and GRACE at discharge scores on MACCEs was consistent between male and female patients (all *p* for interaction >0.05).

To assess whether predictive performance differed depending on sex, primary outcome was also analyzed comparing ${\mathrm{CHA}}_{2}$${\mathrm{DS}}_{2}$-VASc, GRACE at admission and GRACE at discharge in subgroups defined by sex. The cumulative incidence of MACCEs during follow-up was shown in Fig. 3. ${\mathrm{CHA}}_{2}$${\mathrm{DS}}_{2}$-VASc and GRACE at discharge significantly stratified high-risk patients across male and female in a consistent manner (*$p_{\text{interaction}}$* = 0.216 and *$p_{\text{interaction}}$* = 0.088, respectively). In addition, three risk models remained associated with the risk of all-cause death independent of sex category (*$p_{\text{interaction}}$* = 0.982 for ${\mathrm{CHA}}_{2}$${\mathrm{DS}}_{2}$-VASc; *$p_{\text{interaction}}$* = 0.857 for GRACE at admission; *$p_{\text{interaction}}$* = 0.977 for GRACE at discharge). We did not detect any relevant interaction with the predictive values in any of subgroups. The results of subgroup analyses were displayed in **Supplementary Table 3**.

## 4. Discussion

In the present study, we assessed prognostic values of the ${\mathrm{CHA}}_{2}$${\mathrm{DS}}_{2}$-VASc, GRACE at admission and GRACE at discharge scores in adverse outcomes among AF patients with ACS or undergoing PCI during 3-year follow-up. We demonstrated that higher ${\mathrm{CHA}}_{2}$${\mathrm{DS}}_{2}$-VASc and GRACE at discharge scores were independently associated with increased risk of MACCEs, but the GRACE score at admission was not. The fact that ${\mathrm{CHA}}_{2}$${\mathrm{DS}}_{2}$-VASc and GRACE at discharge demonstrated comparable discriminative capacity meanwhile GRACE at admission provided relatively lower discrimination further supported this viewpoint. For prediction of all-cause mortality, three models displayed good discriminative capacity. The GRACE at discharge showed better predictive ability. A significant discrimination improvement of GRACE at discharge compared to the ${\mathrm{CHA}}_{2}$${\mathrm{DS}}_{2}$-VASc was detected. In addition, none of the three scores had an acceptable value in predicting major or minor bleeding during the follow-up.

The co-existence of AF and the need for PCI is a much more complicated situation compared to suffering from AF or CAD alone. Existing evidence reports the incidence of ACS with concomitant AF between 6% to 22%, with an increased incidence in elderly and female patients [17, 18]. AF is a well-established marker of poor short- and long-term prognosis in patients with ACS and is associated with an increased risk of overall mortality. An analysis derived from 1558205 ACS patients observed that patients with AF had significantly longer and more complicated hospital stays with nearly double adjusted in-hospital mortality [19]. Pilgrim *et al*. [20] showed that among patients with CAD undergoing revascularization with drug-eluting stents (DES), AF conferred a rising risk of both all-cause mortality and ischemic stroke during four-year follow-up. Similar results were obtained from sub analysis of the Global Registry of Acute Coronary Events (GRACE) study where ACS patients with concomitant AF were more likely to have a complicated in-hospital course than those without AF [21]. Meanwhile, in a large-scale, prospective registry including 29,679 consecutive patients presenting with AF, a prior ACS conferred higher adjusted risks of stroke, systemic embolism, all-cause mortality and CV mortality [22]. In the present study, we reported that the 3-year incidences of composite MACCEs, all-cause mortality, CV mortality reached 15.6%, 9.7% and 7.0% respectively. In the EPICOR (long-tErm follow-uP of antithrombotic management patterns In acute CORonary syndrome patients) Asia study, 6.2% patients experienced the composite endpoint of death, MI and ischemic stroke and 3.6% suffered all-cause death (including 1.3% cardiovascular-related) within 2 years. Although our analysis was from a different group of patients in EPICOR Asia, it could be predicted that ACS combined with AF had a numerically higher relative risk in long-term adverse events compared to ACS alone. Whether AF contributes to the onset of ACS or if ACS leads to AF is beyond the scope of this paper as we lack the precise information about the time of appearance of these diseases. However, previous studies observed that AF could promote inflammation that could cause a prothrombotic state and eventually coronary artery occlusion [23]. In addition, AF with high heart ventricular rates might yield symptoms of myocardial ischemia characterized by an imbalance between demand and blood supply [24]. Conversely, CAD affecting the atrial branches could result atrial scarring and remodeling to form a substrate conducive for consequent persistent AF [25]. In the past three decades, catheter ablation has evolved to a well-established treatment option for AF patients to obtain rhythm control. The safety and effectiveness of ablation in increasing freedom from recurrences and lowering AF burden during one year follow-up has been documented in multiple clinical trials [26, 27]. While the study lacked information on catheter ablation, there is no randomized controlled trial sufficiently large to properly evaluate a reduction in thromboembolic events compared with antiarrhythmic dugs [5].

The ${\mathrm{CHA}}_{2}$${\mathrm{DS}}_{2}$-VASc score has been widely used for the assessment of thrombo-embolic risk and guiding antithrombotic therapy in AF or atrial flutter. A growing number of studies have assessed the predictive accuracy of ${\mathrm{CHA}}_{2}$${\mathrm{DS}}_{2}$-VASc score in patients with CAD. Podolecki *et al*. [28] included 2647 consecutive acute myocardial infarction (AMI) patients without AF and found that the risk of ischemic stroke and all-cause death in ${\mathrm{CHA}}_{2}$${\mathrm{DS}}_{2}$-VASc ≥4 increased 4-fold compared to ${\mathrm{CHA}}_{2}$${\mathrm{DS}}_{2}$-VASc = 1. Besides, every point in ${\mathrm{CHA}}_{2}$${\mathrm{DS}}_{2}$-VASc score was independently associated with 41% increase in stroke risk and 23% increase in mortality. Both ${\mathrm{CHADS}}_{2}$ and ${\mathrm{CHA}}_{2}$${\mathrm{DS}}_{2}$-VASc scores were evaluated in a study of 929 AF patients referred for PCI. A high ${\mathrm{CHA}}_{2}$${\mathrm{DS}}_{2}$-VASc score was predictive of all-cause mortality and MACCE at 12-months while the ${\mathrm{CHADS}}_{2}$ score could only predict MACCEs. ${\mathrm{CHADS}}_{2}$ and ${\mathrm{CHA}}_{2}$${\mathrm{DS}}_{2}$-VASc were not associated with major bleeding [29]. These findings were further supported by another survey enrolling 13,422 ACS patients which demonstrated that a higher ${\mathrm{CHA}}_{2}$${\mathrm{DS}}_{2}$-VASc score was associated with an increased risk of 1-year mortality after adjusting for in-hospital treatments [30]. However, the available studies mainly targeted ACS or ACS undergoing PCI as research population and there is little evidence evaluating the association between risk models and stable coronary artery disease (SCAD) patients undergoing elective PCI. Also, previous study often calculated c-statistics or utilized Kaplan-Meier curves to illustrate the predictive performance of ${\mathrm{CHA}}_{2}$${\mathrm{DS}}_{2}$-VASc score. There are no sufficient data regarding the impact of post-discharge antithrombotic regimens as it plays an important role in long-term outcomes. In the current study, we included 433 (30.8%) SCAD patients who were eligible for elective PCI, among whom 54 suffered MACCE and 18 died within 3 years. We found that ${\mathrm{CHA}}_{2}$${\mathrm{DS}}_{2}$-VASc score had a good predictive performance in MACCE (c-statistic: 0.677 as continuous) and all-cause death (c-statistics: 0.750 as continuous) throughout the entire range of the score. Of note, the thromboembolic risk was approximately 1.184 times greater for each point increase in the ${\mathrm{CHA}}_{2}$${\mathrm{DS}}_{2}$-VASc score, while low and intermediate category reclassified by ${\mathrm{CHA}}_{2}$${\mathrm{DS}}_{2}$-VASc had comparable risk of MACCEs after adjusting for covariates. These findings could not be attributed to poor predictive performance for the cutoffs were chosen on the basis of previous literature subjectively. It is the first to consider not only clinical presentations and laboratory results but also post-discharge medications as potential risk factors. Patients who experienced MACCE were less likely to be prescribed Aspirin and Ticagrelor but more frequently to receive anticoagulant therapy at discharge. Multivariable Cox regression analysis including antithrombotic regimens as covariates showed that ${\mathrm{CHA}}_{2}$${\mathrm{DS}}_{2}$-VASc score was an independent risk factor for MACCEs and all-cause mortality. Besides, ${\mathrm{CHA}}_{2}$${\mathrm{DS}}_{2}$-VASc >4 could increase the risk of MACCEs by double and all-cause death by 5 times compared to ${\mathrm{CHA}}_{2}$${\mathrm{DS}}_{2}$-VASc equal to 1 or 2.

The GRACE ACS score was derived from an international registry of ACS patients and has been a well-recognized risk system to stratify patients according to their estimated risk of future death or ischemic events. Several studies have also tried to evaluate and compare the predictive performance of ${\mathrm{CHA}}_{2}$${\mathrm{DS}}_{2}$-VASc and GRACE scores to determine which system is more suitable for AF patients combined with ACS or coronary stenting. Fauchier *et al*. [31] aimed to find out the most appropriate score among ${\mathrm{CHA}}_{2}$${\mathrm{DS}}_{2}$-VASc, GRACE at admission, REACH [32], SYNTAX [33] and Anatomical and Clinical Syntax II Score (ACSS) [34] to use in the setting of 845 AF with coronary stenting. The results indicated that ${\mathrm{CHA}}_{2}$${\mathrm{DS}}_{2}$-VASc was the best predictor of stroke and thromboembolic events with a c-statistics of 0.604 and SYNTAX was better to predict MACE with a c-statistics of 0.612. GRACE at admission was the best to predict all-cause mortality with a c-statistics of 0.682 [31]. Another retrospective study consisting of 1452 consecutive patients undergoing PCI with a diagnosis of AF demonstrated that the GRACE at admission but not the ${\mathrm{CHA}}_{2}$${\mathrm{DS}}_{2}$-VASc score was associated with the incidence of MACEs within 1 year. However, the two scores showed similar predictive performance in the prediction of all-cause mortality [35]. Although the above researches came to controversial conclusions, they adopted GRACE score designed for predicting cumulative six month risk of death or MI other than the GRACE score developed for predicting post-discharge outcomes. The variables used by GRACE at discharge involved history of CHF, history of MI and in-hospital PCI suggesting that more weighting is given to chronic conditions in the post-discharge system. To the best of our knowledge, this is the first report comparing ${\mathrm{CHA}}_{2}$${\mathrm{DS}}_{2}$-VASc, GRACE at admission and GRACE at discharge scores in the same cohort. We found that the ${\mathrm{CHA}}_{2}$${\mathrm{DS}}_{2}$-VASc and GRACE at discharge scores showed significant prognostic values in long-term MACCEs according to multivariable analysis as well as c-statistics, while the GRACE at admission had no impact on predicting MACCEs. Notably, the prognostic ability of GRACE at discharge score could be weakened after grouping in accordance with recommendations of the GRACE system. On the other hand, GRACE at discharge demonstrated significant superiority in predicting all-cause mortality with a c-statistics of 0.846 (as continuous) to ${\mathrm{CHA}}_{2}$${\mathrm{DS}}_{2}$-VASc score with a c-statistics of 0.750 (*p *< 0.001). The results were consistent with the differences of purpose when the two risk scoring systems were established. The ${\mathrm{CHA}}_{2}$${\mathrm{DS}}_{2}$-VASc score was developed for AF in stratifying high-risk patients who would sustain thromboembolic events. Nevertheless, the main outcome measured in designing GRACE at discharge score was all-cause mortality during 6 months follow-up. As discussed above, any risk score has to balance simplicity and practicality against precision. In the present study, the GRACE at discharge score should undoubtedly be advocated for evaluating the long-term survival if conditions permit. The ${\mathrm{CHA}}_{2}$${\mathrm{DS}}_{2}$-VASc only performed modestly in predicting all-cause mortality, but it could be utilized rapidly if biomarkers or electrocardiogram information necessary for calculating the GRACE were difficult to obtain. Furthermore, it was suitable to combine ${\mathrm{CHA}}_{2}$${\mathrm{DS}}_{2}$-VASc with GRACE at discharge to improve the accuracy of risk stratification, leading to more effective clinical decision-making and prolonged survival of AF patients with ACS or undergoing PCI.

The following were several limitations in the present study. First, this is an observational, prospective, single-center registry and has its inherent residual confounding bias. Our findings should be carefully interpreted when applied to external validation cohorts. However, we analyzed a total of 1408 AF patients with ACS or undergoing PCI and the sample size was comparable to those of similar researches. Second, we did not assess the prognostic values of the three models according to the subtypes of AF for we were unable to determine the accurate order of the presence of AF and ACS. Previous evidence suggested that only permanent AF was an independent predictor for death in AMI patients treated invasively [36]. Third, the post-discharge antithrombotic regimens were collected and treated as a covariate in our study. During 3-year follow-up, the medication adherence of participants and the possible transitions in dual-antiplatelet therapy after coronary stenting were unable to be obtained. The changes in anticoagulant or antiplatelet therapies might have significantly affected the incidence of ischemic or bleeding events. Further well-designed clinical trials are needed to compare and validate the prediction performance of several risk stratification systems for AF patients with ACS or undergoing stent implantation.

## 5. Conclusions

In the setting of coexistence of AF and ACS or coronary stenting, higher ${\mathrm{CHA}}_{2}$${\mathrm{DS}}_{2}$-VASc and GRACE at discharge score were independently associated with increased risk of MACCEs and they had comparable discriminative capacities. Both ${\mathrm{CHA}}_{2}$${\mathrm{DS}}_{2}$-VASc and GRACE at discharge scores demonstrated good prognostic values in all-cause mortality. A significant discrimination improvement of GRACE at discharge was detected compared to ${\mathrm{CHA}}_{2}$${\mathrm{DS}}_{2}$-VASc. The GRACE at admission score could not identify patients at high risk of MACCEs. Further studies are needed to validate the clinical significance of these scores externally or help build a more accurate and practical risk score.

## Supplementary Material

Supplementary material associated with this article can be found, in the online version, at https://doi.org/10.31083/j.rcm2305168.
