# Supplementary material for: Clinical Application of CHA2DS2-VASc versus GRACE Scores for Assessing the Risk of Long-term Ischemic Events in Atrial Fibrillation and Acute Coronary Syndrome or PCI
Source: Rev Cardiovasc Med. 2022 May 11;23(5):168. doi: 10.31083/j.rcm2305168 (PMC11273896; doi:10.31083/j.rcm2305168)
Supplement: Supplementary file 1 [file 2153-8174-23-5-168-s1.zip › 2153-8174-23-5-168-s1.docx]

Supplementary Table 1. Distribution of CHA_2_DS_2_-VASc score in the study population.

| Variable | Total (n = 1408) | MACCE | | *p* value | All-cause death | | *P* value |
| --- | --- | --- | --- | --- | --- | --- | --- |
|  |  | No (n = 1188) | Yes (n = 220) |  | No (n=1267) | Yes (n = 141) |  |
| CHA_2_DS_2_-VASc, n (%) |  |  |  | <0.001 |  |  | <0.001 |
| 1 | 148 (10.5) | 141(11.9) | 7 (3.2) |  | 145 (11.4) | 3 (2.2) |  |
| 2 | 232 (16.5) | 210 (17.7) | 22 (10.0) |  | 227 (17.8) | 5 (3.7) |  |
| 3 | 325 (23.1) | 289 (24.3) | 36 (16.4) |  | 307 (24.1) | 18 (13.2) |  |
| 4 | 259 (18.4) | 216 (18.2) | 43 (19.5) |  | 234 (18.4) | 25 (18.4) |  |
| 5 | 213 (15.1) | 166 (14.0) | 47 (21.4) |  | 184 (14.5) | 29 (21.3) |  |
| 6 | 122 (8.7) | 91 (7.7) | 31 (14.1) |  | 98 (7.7) | 24 (17.6) |  |
| 7 | 74 (5.3) | 51(4.3) | 23 (10.5) |  | 55 (4.3) | 19 (14.0) |  |
| 8 | 26 (1.8) | 16 (1.3) | 10 (4.5) |  | 16 (1.3) | 10 (7.4) |  |
| 9 | 9 (0.6) | 8 (0.7) | 1 (0.5) |  | 6 (0.5) | 3 (2.2) |  |

Supplementary Table 2. Predictive ability of the CHA_2_DS_2_-VASc and GRACE scores (continuous or categorical) for the detection of MACCE or all-cause mortality.

|  | C-statistic | 95% CI | *p* | NRI,% | 95%CI |
| --- | --- | --- | --- | --- | --- |
| MACCE (continuous) |  |  |  |  |  |
| CHA_2_DS_2_-VASc | 0.677 | 0.637–0.717 | Reference | Reference |  |
| GRACE (in-hospital) | 0.629 | 0.585–0.673 | 0.041 | -13.21 | (-21.60, -7.38) |
| GRACE (post-discharge) | 0.699 | 0.659–0.740 | 0.281 | 5.48 | (-14.42, 32.63) |
| MACCE (categorical) |  |  |  |  |  |
| CHA_2_DS_2_-VASc | 0.653 | 0.614–0.692 | Reference | Reference |  |
| GRACE (in-hospital) | 0.609 | 0.567–0.650 | 0.058 | -16.70 | (-49.93,4.56) |
| GRACE (post-discharge) | 0.653 | 0.614–0.693 | 0.993 | -0.87 | (-41.55,26.57) |
| All-cause mortality (continuous) |  |  |  |  |  |
| CHA_2_DS_2_-VASc | 0.750 | 0.705–0.794 | Reference | Reference |  |
| GRACE (in-hospital) | 0.775 | 0.732–0.818 | 0.310 | 5.57 | (-19.5,33.63) |
| GRACE (post-discharge) | 0.846 | 0.813–0.880 | <0.001 | 45.13 | (17.37,72.31) |
| All-cause mortality (categorical) |  |  |  |  |  |
| CHA_2_DS_2_-VASc | 0.716 | 67.32–75.81 | Reference | Reference |  |
| GRACE (in-hospital) | 0.744 | 70.34–78.43 | 0.25 | 7.58 | (-2.85,46.74) |
| GRACE (post-discharge) | 0.786 | 75.28–81.96 | 0.003 | 15.37 | (6.10,31.45) |

Supplementary Table 3. The predictive performance of CHA_2_DS_2_-VASc and GRACE scores on primary outcome or all-cause mortality stratified by sex category.

Part A. Cumulative incidence and hazard ratios of CHA_2_DS_2_-VASc score.

| CHA_2_DS_2_-VASc | Male (n = 1027) | | | Female (n = 381) | | | P for interaction |
| --- | --- | --- | --- | --- | --- | --- | --- |
|  | 1,2 (n = 353) | 3,4 (n = 445) | >4 (n = 229) | 1,2 (n = 27) | 3,4 (n = 139) | >4 (n = 215) |  |
| **MACCE** |  |  |  |  |  |  | 0.216 |
| Number of events (%) | 7.08 | 12.13 | 25.33 | 14.81 | 17.99 | 25.12 |  |
| Adjusted HR (95% CI) | 1.00 (reference) | 1.310 (0.799–2.149) | 2.176 (1.285–3.685) | 1.00 (reference) | 1.646 (0.858–3.156) | 2.053 (1.063–3.964) |  |
| **Death** |  |  |  |  |  |  | 0.982 |
| Number of events (%) | 2.27 | 7.64 | 19.65 | 0 | 6.47 | 18.60 |  |
| Adjusted HR (95% CI) | 1.00 (reference) | 2.507 (1.095–5.740) | 4.399 (1.900–10.187) | 1.00 (reference) | 2.573 (0.745–8.888) | 7.559 (2.311–24.731) |  |

Part B. Cumulative incidence and hazard ratios of GRACE at admission score

| GRACE at admission | Male (n = 1027) | | | Female (n = 381) | | | P for interaction |
| --- | --- | --- | --- | --- | --- | --- | --- |
|  | ≤108 (n = 407) | 109-140 (n = 381) | >140 (n = 239) | ≤108 (n = 108) | 109-140 (n = 136) | >140 (n = 137) |  |
| **MACCE** |  |  |  |  |  |  | 0.207 |
| Number of events (%) | 9.58 | 11.29 | 23.01 | 16.67 | 19.12 | 28.47 |  |
| Adjusted HR (95% CI) | 1.00 (reference) | 1.013 (0.642–1.598) | 1.533 (0.796–2.952) | 1.00 (reference) | 1.212 (0.641–2.290) | 1.178 (0.484–2.870) |  |
| **Death** |  |  |  |  |  |  | 0.857 |
| Number of events (%) | 1.97 | 7.09 | 21.76 | 2.78 | 6.62 | 27.01 |  |
| Adjusted HR (95% CI) | 1.00 (reference) | 3.026 (1.348–6.789) | 5.632 (2.223–14.273) | 1.00 (reference) | 1.987 (0.520–7.589) | 4.090 (0.919–18.208) |  |

Part C. Cumulative incidence and hazard ratios of GRACE at discharge score

| GRACE at discharge | Male (n = 1027) | | | Female (n = 381) | | | P for interaction |
| --- | --- | --- | --- | --- | --- | --- | --- |
|  | ≤88 (n = 324) | 89-118 (n = 395) | >118 (n = 308) | ≤108 (n = 84) | 109-140 (n = 141) | >140 (n = 156) |  |
| **MACCE** |  |  |  |  |  |  | 0.088 |
| Number of events (%) | 7.72 | 10.13 | 23.38 | 13.09 | 20.57 | 27.56 |  |
| Adjusted HR (95% CI) | 1.00 (reference) | 1.249 (0.757–2.063) | 3.235 (2.048–5.108) | 1.00 (reference) | 1.722 (0.858–3.453) | 2.544 (1.309–4.946) |  |
| **Death** |  |  |  |  |  |  | 0.977 |
| Number of events (%) | 0.93 | 4.81 | 21.10 | 1.19 | 5.67 | 25.64 |  |
| Adjusted HR (95% CI) | 1.00 (reference) | 4.028 (1.183–13.707) | 10.006 (3.015–33.208) | 1.00 (reference) | 3.567 (0.440–28.879) | 10.220 (1.305–80.021) |  |

**Notes**: Adjusted for emergency presentation, atrial fibrillation patterns, subtypes of coronary artery disease, anticoagulant therapy, use of aspirin, use of ticagrelor.
